# Cost Drivers for Voluntary Medical Male Circumcision Using Primary Source Data from Sub-Saharan Africa

**DOI:** 10.1371/journal.pone.0084701

**Published:** 2014-05-06

**Authors:** Lori Bollinger, Adebiyi Adesina, Steven Forsythe, Ramona Godbole, Elan Reuben, Emmanuel Njeuhmeli

**Affiliations:** 1 Health Policy Initiative Costing Task Order, Washington, District of Columbia, United States of America; 2 Health Policy Project, Washington, District of Columbia, United States of America; 3 Futures Institute, Glastonbury, Connecticut, United States of America; 4 Futures Group, Washington, District of Columbia, United States of America; 5 United States Agency for International Development, Washington, District of Columbia, United States of America; World Health Organization, Switzerland

## Abstract

**Background:**

As voluntary medical male circumcision (VMMC) programs scale up, there is a pressing need for information about the important cost drivers, and potential efficiency gains. We examine those cost drivers here, and estimate the potential efficiency gains through an econometric model.

**Methods and Findings:**

We examined the main cost drivers (i.e., personnel and consumables) associated with providing VMMC in sub-Saharan Africa along a number of dimensions, including facility type and service provider. Primary source facility level data from Kenya, Namibia, South Africa, Tanzania, Uganda, and Zambia were utilized throughout. We estimated the efficiency gains by econometrically estimating a cost function in order to calculate the impact of scale and other relevant factors. Personnel and consumables were estimated at 36% and 28%, respectively, of total costs across countries. Economies of scale (EOS) is estimated to be eight at the median volume of VMMCs performed, and EOS falls from 23 at the 25th percentile volume of VMMCs performed to 5.1 at the 75th percentile.

**Conclusions:**

The analysis suggests that there is significant room for efficiency improvement as indicated by declining EOS as VMMC volume increases. The scale of the fall in EOS as VMMC volume increases suggests that we are still at the ascension phase of the scale-up of VMMC, where continuing to add new sites results in additional start-up costs as well. A key aspect of improving efficiency is task sharing VMMC procedures, due to the large percentage of overall costs associated with personnel costs. In addition, efficiency improvements in consumables are likely to occur over time as prices and distribution costs decrease.

## Introduction

Voluntary medical male circumcision (VMMC) services are an important tool in the arsenal of weapons being used to fight the HIV/AIDS epidemic. The World Health Organization (WHO) and the Joint United Nations Programme on HIV/AIDS (UNAIDS) recommend VMMC as an important component of the HIV prevention portfolio in countries with generalized epidemics that have low male circumcision prevalence and high HIV prevalence, where basic program activities are those that have a direct effect on reducing the transmission of HIV [1]. Randomized controlled trials in Uganda [2], Kenya [3], and South Africa [4] showed that the probability of HIV transmission is reduced by 60% for men who are circumcised, making it a highly effective intervention. Investments for this intervention are not trivial, but could result in significant overall cost savings—a recent study estimated that scaling up VMMC in 13 countries in sub-Saharan Africa to reach 80% of adult men would cost US$2 billion, but would save US$16.5 billion in treatment costs [5].

Due to the size of the resources required, recent studies have examined potential cost savings through delivering VMMC via different service delivery modes, utilizing task-shifting approaches, as well as employing non-surgical devices in place of the conventional surgical circumcision. A recent study in Tanzania found that there were no significant differences in unit cost (and therefore no savings) between non-campaign and campaign service delivery models. The non-campaign model was estimated at US$45.38 per circumcision, while campaign service delivery was assessed at US$45.98 with labor and consumables making up approximately 70% of the costs [6]. Studies have shown that task shifting of VMMC services does not increase the number of adverse events reported [7], [8] and may actually result in significant cost savings. In Uganda, shifting the procedure from surgeons to medical officers resulted in a savings of 24% [9], while in Namibia shifting the procedure from physicians to surgical nurses reduced the average unit cost by 31% [10]. Finally, two recent studies found that using the alternative non-surgical devices, Shang Ring [11] or PrePex [12], did not result in significant direct cost savings as compared to surgical circumcision.

Econometric analyses can contribute to identifying potential cost savings associated with delivering HIV services. Various methodologies have been utilized to estimate potential efficiency gains for HIV prevention, including estimating efficiency frontiers [13], [14] and using a generalized linear mixed model to estimate the effect of cost determinants on annual per-patient HIV treatment costs [15]. We build on a recent study in estimating a cost function for HIV prevention services to calculate the potential economies of scale (EOS) associated with VMMC as well as the impact of other factors [16]. With the increasing importance of and investment in VMMC, it is important to understand the main cost drivers associated with providing VMMC services, and also any possible efficiency gains that might be achieved by adapting the service provision to each country setting. Results of this analysis could assist countries in planning scale-up of VMMC service delivery.

## Methods

This analysis employed primary source facility-level data from Nyanza, Kenya (29 facilities), Namibia (8 facilities), South Africa (9 facilities), Tanzania (18 facilities), Uganda (26 facilities), and Zambia (9 facilities), for a total of 99 facilities (see [5], [6], [9] for detailed discussion of the data including sampling approaches, which varied by country). Data were collected between 2008 and 2011 with samples stratified by geographic region, urban/rural designation, service provider (nongovernmental organization [NGO], public, private), type of facility (hospital, health center, dispensary), and service delivery mode (outreach, campaign, fixed/static). Data collection periods varied among countries. For facilities that were operational for only part of the year or for which data were collected for only part of the year, costs were annualized to allow for comparison across countries. Data collected included programmatic and non-financial operational data, such as service delivery mode (i.e., fixed/static, outreach, mobile) and number of clients served per period of time; direct costs, including consumables, reusables, personnel and training costs; and indirect costs, including support service costs like central support/management staff, international consultants, maintenance and supervisory workers, insurance, utilities/telephone, office furniture, other equipment such as autoclaves and typewriters, vehicle maintenance, other electronic maintenance [17], [18]. Costs associated with demand creation including mobilization or promotional activities were not included because these data were not collected in all countries, and were defined inconsistently in the countries where the data were obtained.

In order to be able to compare data across countries, as well as analyze common characteristics, we inflated all cost data to 2012 US dollars by using the appropriate year of the US gross domestic product (GDP) deflator [19], and then adjusted to a regionalized cost by multiplying each input cost by the ratio of the country-level purchasing-power-parity-adjusted (PPP-adjusted) gross national income (GNI) per capita to the same regional-level variable for sub-Saharan Africa [20].

We then utilized these data in two ways: first, we analyzed the data using general descriptive statistical methods, computing means and medians for continuous variables and frequency counts and percentages for categorical variables. Second, we performed econometric analyses to assess potential efficiency gains by estimating a cost function where total VMMC costs are a function of input costs and quantity produced (i.e., the number of male circumcisions performed) in addition to other variables that may affect output, including male circumcision prevalence, urban/rural designation, service delivery mode, and service provider category. We assumed that the cost function is well-behaved mathematically (i.e., linearly homogeneous in input prices), and that facilities behave in a cost-minimizing way, such that the cost function is:
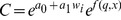
(1)where: C = average total cost; a_0_ = constant; a_i_ = coefficients associated with i input prices; w_i_ = vector of inputs; q = quantity produced; and x = vector of independent variables that might shift the cost function. Taking the logarithm of both sides and including linear, squared, and cubed output variables in the specification results in the following equation to be estimated:
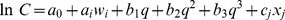
(2)where b_i_ are the coefficients associated with the three output variables. In order to derive a measure of EOS [21], [22], we follow [16], [21] in calculating the marginal cost (MC, or 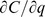) is calculated by differentiating C in equation (2) with respect to q:
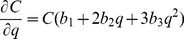
(3)


The EOS measure, which is an indication of efficiency, can then be calculated as:
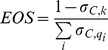
(4)where 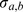 is the elasticity of a with respect to b, and k is capital stock. Note that 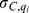 is equal to the product of the marginal cost of output, equation (3), and the ratio of output to total cost. Thus here, where we control for variations in capital stock and we have only one output q, the equation to calculate economies of scale becomes:
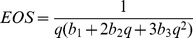
(5)


When EOS equals one, there are constant returns to scale, and doubling the inputs results in a doubling of the outputs. When EOS is greater than one, the level of output is less efficient than the level of output achieved in the presence of constant returns to scale. When EOS is less than one, the level of output is more efficient.

Descriptive statistics are displayed in Table 1 for the variables used in the econometric estimation, including the time period over which data were collected. As discussed above, all cost data have been transformed to 2012 US dollars, and are adjusted to a common regional level using relative PPP-adjusted GNI per capita data, so that they can be compared directly. Note that not all categories of data were collected for all countries; for example, training costs were not collected separately in Namibia, South Africa, or Zambia. Because of this, we made two adjustments in the independent variables that we use. First, with respect to labor-related costs, instead of using separate independent variables for labor-related costs (direct personnel, training, support, and management/supervisory costs), we calculated a variable for total labor costs, which is the sum of these four costs. Second, because excluding the missing observations for capital and reusable costs would cut the sample in half (including completely excluding Namibia, Kenya, and Zambia), and capital and reusable costs are such a small proportion of total costs, we excluded these variables from the analysis. A dummy variable was used for each country to allow for the impact of these excluded variables, as well as other country-specific effects. In addition, we performed a sensitivity analysis to assess the impact of capital cost on marginal cost and EOS using data for the three countries where capital cost data were collected (Kenya, South Africa and Uganda). The marginal cost for the three countries when capital cost was included in the regression was $3.72 and when capital was excluded, the marginal cost was $3.51, a difference of about 5%. Similarly, the EOS for the three countries with capital cost included in the regression was 11.97 and when capital cost was excluded, $11.99, an even smaller difference. The negligible difference between the inclusion and exclusion of capital cost suggests its relatively low contribution to total cost.

**Table 1:** Descriptive statistics for regression variables by country: Averages and (standard deviations).

	Average of sample	Kenya (Nyanza)	Namibia	South Africa	Tanzania	Uganda	Zambia
Time period of data collection		March 2010	April–May 2008	April 1, 2008–Mar 31, 2009	2010 and 2011	June–July 2009	2010
Number of facilities (n)	99	29	8	9	18	26	9
Number of male circumcisions per facility (q)	750	734	35	3,828	1,914	286	308
Unit cost data (2012 US$)							
**Direct Costs per circumcision**							
Consumables	$13.89	$10.30	$15.08	$6.44	$20.67	$15.09	$11.04
(standard deviation)	($6.35)	($5.49)	($3.69)	($1.32)	($4.56)	($3.87)	($5.24)
Reusable Supplies	$0.19	$0.00	$0.06	$0.01	$0.16	$0.35	$0.00
(standard deviation)	($0.14)	n/a	($0.03)	($0.02)	($0.08)	($0.00)	n/a
Personnel	$17.55	$14.54	$14.79	$3.19	$31.03	$8.26	$19.83
(standard deviation)	($13.41)	($14.00)	($13.59)	($2.82)	($10.02)	($6.57)	($18.63)
Training	$5.96	$2.44	$0.00	$0.00	$8.49	$2.10	$0.00
(standard deviation)	($11.90)	($3.88)	n/a	n/a	($22.60)	($6.21)	n/a
**Indirect Costs per circumcision**							
Capital Costs	$4.91	$2.78	$0.00	$1.09	$2.87	$1.03	$13.99
(standard deviation)	($12.50)	($1.97)	n/a	($0.44)	($13.95)	($1.31)	($25.36)
Maintenance and Utilities	$3.47	$3.55	$0.72	$5.29	$4.36	$0.58	$11.30
(standard deviation)	($8.13)	($3.88)	($1.16)	($1.81)	($15.75)	($1.86)	($11.18)
Support Personnel	$2.62	$3.56	$0.70	$6.35	$1.50	$2.22	$3.72
(standard deviation)	($2.70)	($2.39)	($0.52)	($1.72)	($2.31)	($3.07)	($2.76)
Management and Supervision	$0.65	$1.16	$0.04	$0.00	$0.77	$0.36	$1.33
(standard deviation)	($1.34)	($1.61)	($0.06)	n/a	($1.84)	($0.53)	($1.63)
Total Unit Cost	$49.17	$38.33	$31.38	$22.37	$69.85	$30.00	$61.21
(standard deviation)	($28.50)	($17.31)	($16.25)	($3.89)	($36.84)	($11.66)	($22.24)
Other descriptive variables (proportions)							
Setting (excluded category: Rural)							
Urban = 1	0.58	0.52	1.00	0.33	0.50	0.65	0.33
Service provider (excluded category: Public)							
NGO = 1	0.36	1.00	0.13	0.11	0.00	0.15	0.11
Private = 1	0.10	0.00	0.00	0.00	0.00	0.35	0.11
Facility type (excluded category: Health Centers)							
Dispensary = 1	0.06	0.21	0.00	0.00	0.00	0.00	0.00
Hospital = 1	0.67	0.48	1.00	0.78	0.89	0.65	0.44
Service delivery mode (excluded category: Fixed/Static)							
Outreach = 1	0.16	0.52	0.00	0.00	0.06	0.00	0.00
Campaign = 1	0.06	0.00	0.00	0.00	0.33	0.00	0.00
Male circumcision prevalence	0.31	0.45	0.16	0.33	0.34	0.25	0.09
(standard deviation)	(0.11)	n/a	(0.09)	(0.08)	(0.05)	(0.17)	(0.04)

All costs are in 2012 US$ and adjusted to the sub-Saharan Africa region using the ratio of the country PPP-adjusted GNI per capita to the region PPP-adjusted GNI per capita.

We used several variables to control for possible shifts in the cost function. The source for these data is the primary source data collection described above, with the exception of male circumcision prevalence data, which are from Demographic and Health Surveys. We assumed, *a priori*, that costs would be higher in (1) urban settings (relative to rural settings, the excluded category); (2) NGO and private service providers (relative to public service providers, the excluded or reference category); (3) hospitals (relative to health centers, the excluded category)—although dispensaries are likely to be a lower cost facility, because only Kenya reported data for that type of facility, we did not use it as the excluded category for facility type; and (4) outreach and campaign service delivery modes (relative to fixed/static service delivery modes, the excluded category).

Finally, the *a priori* expectation regarding the effect of male circumcision prevalence on cost is not clear. Male circumcision prevalence may be associated with lower unit costs if minimum efficient scale has not yet been reached; however, the association may be positive if scaling up VMMC has resulted in reaching beyond minimum efficient scale. We used Stata version 12 to perform the econometric estimation using a generalized linear model (GLM), correcting for heteroskedasticity by calculating robust standard errors using the Huber-White Sandwich estimator [23]; the actual Stata command used was: glm depvar indepvars, link(log) family (gamma) vce(robust).

## Results

### Descriptive results

Overall, the average total cost per facility (C) across all 99 facilities was US$49.17, with an average of 750 male circumcisions performed annually (q) per facility. Within the average unit cost , personnel costs account for the greatest amount at US$17.55 (36%), followed by consumables (US$13.89) (28%), training costs (US$5.96) (12%), capital costs (US$4.91) (10%), maintenance and utilities (US$3.47) (7%), support personnel (US$2.62) (5%), management and supervision costs (US$0.65) (1%), and finally reusable supplies (US$0.19) (<1%). Note that supply chain costs were not gathered as part of these costing exercises; a recent study found that supply chain costs (excluding the consumables) was, on average, US$10.93 [24]. After adjustment to the regional-level GNI per capita, the range of average unit costs of VMMC by country varied from a high of US$70 in Tanzania to a low of US$22 in South Africa; this relatively lower cost in South Africa is discussed further below.

Across all 99 facilities, 58% are in urban areas, with the highest proportion of urban facilities in the Namibia sample (eight of eight, or 100%) and the lowest proportion in Zambia and South Africa (three of nine, or 33%). In this sample, slightly over half of VMMCs were performed by public providers (with 100% of VMMCs in Tanzania performed by public providers); 36% were provided by NGOs (with 100% of the Kenya sample being NGO providers), and only 10% were provided by private providers (with the highest percentage, 35%, observed in Uganda). Overall, two-thirds of the VMMCs in these samples were performed in hospitals; the rest were performed in health centers (with the exception of several dispensaries in Kenya, as noted above). The vast majority of VMMCs (78%) were performed in fixed/static sites; service delivery through outreach was utilized in Kenya (Nyanza) and Tanzania, and only Tanzania included VMMC campaigns. Finally, the regional male circumcision prevalence rates associated with the various facilities averaged 31%, ranging from a low of 9% for the nine facilities in Zambia to 45% for the 29 facilities in Nyanza, Kenya.

We further analyzed the unit cost data by examining the percentage each component contributed to total unit cost according to a number of characteristics (see Table 2; note that the first row repeats the PPP-adjusted unit cost displayed at the country level in Table 1, including the PPP-adjusted unit cost according to the various characteristics). Direct personnel costs accounted for the greatest proportion of total unit cost, at 36%. This proportion varied across countries: Namibia and Tanzania had relatively higher proportions of direct personnel costs relative to the total, at 47% and 44%, respectively, followed by Kenya (38%), Zambia (32%), Uganda (28%), and South Africa (14%). Note that the high percentage devoted to direct personnel costs in Namibia continued to be high (47%) even after the non-surgical tasks were shifted away from physicians, resulting in the unit cost reduction of 31% mentioned above [10]; there is not much potential for further task shifting in Namibia. Namibia's unit cost would have presumably been even smaller, but the low volume of clients at each facility caused Namibia to still maintain a relatively high unit cost compared to the other countries. The lowest percentage (14%) devoted to direct personnel costs was found in South Africa, most likely due to the fact that, unlike other countries included in our sample, South Africa has adopted most of the considerations (with the exception of task shifting) recommended by and included in the WHO “Considerations for Implementing Models for Optimizing the Volume and Efficiency (MOVE) of Male Circumcision Services” [25].Two serial cross-sectional surveys of VMMC sites were conducted in Kenya, Republic of South Africa, Tanzania and Zimbabwe in 2011 and 2012. Trained clinicians observed the quality of surgical technique and timed nine steps in the VMMC procedure. Four elements of efficiency (task shifting, task sharing [of suturing], rotation among multiple surgical beds, and use of electrocautery) and quality of surgical technique were assessed as explanatory variables. The data showed time savings from task sharing in suturing and use of electrocautery in South Africa and Zimbabwe (where task shifting was not authorized) [26]. SYMMACS data confirm the efficiency benefits of task sharing of suturing and use of electrocautery [27] already recommended by WHO [25]. Note that the contribution of training costs to unit cost at the facility level was approximately 12% across all of the countries, with three countries having non-zero observations: Kenya (6%), Tanzania (12%), and Uganda (7%). The definition of the training costs included varied by country; for Tanzania and Uganda, training costs were gathered at the facility level, and pertained to training specifically performed for VMMC. In Kenya, although no training cost data were gathered at the facility level, various assumptions were made to calculate the contribution of VMMC-specific training to overall unit cost. In South Africa, a national-level VMMC-specific cost was estimated as part of the total cost of the national program, but was not included in the facility-level unit cost. In Namibia, only in-service training costs were included in the overall unit cost, while in Zambia, no training costs were included.

**Table 2:** Components of unit costs, by country and selected characteristics.

		Countries						Urban/Rural		Service Provider			Type of Facility			Service Delivery Mode		
	Average of countries	Kenya	Namibia	South Africa	Tanzania	Uganda	Zambia	Urban	Rural	NGO	Public	Private	Hospital	Health Center	Dispensary	Outreach	Campaign	Fixed/Static
Actual unit cost (2012 US$, PPP-adjusted)	$49	$38	$31	$22	$70	$30	$61	$47	$57	$36	$65	$26	$53	$47	$55	$55	$70	$45
Direct Costs per circumcision (% of total)																		
Consumables	28.3	26.9	48.0	28.8	29.8	50.3	18.0	32.3	25.9	28.0	29.8	48.8	30.8	28.1	24.6	22.9	23.9	33.3
Reusable Supplies	0.4	0.0	0.2	0.1	0.2	1.2	0.0	0.2	0.3	0.1	0.3	1.3	0.2	0.2	0.0	0.0	0.3	0.2
Personnel	35.7	37.9	47.1	14.2	44.8	27.5	32.4	37.7	43.8	33.1	43.4	33.9	41.9	30.9	50.6	46.5	44.4	37.3
Training	12.1	6.4	0.0	0.0	11.9	7.0	0.0	8.2	10.8	4.9	11.2	7.1	10.6	5.9	3.5	3.5	11.6	9.3
Indirect Costs per circumcision (% of total)																		
Capital Costs	10.0	7.2	0.0	4.9	4.2	3.4	22.9	4.7	7.2	7.2	5.1	2.8	5.2	6.6	6.6	9.9	3.8	5.7
Maintenance and Utilities	7.0	9.3	2.3	23.7	6.4	1.9	18.5	9.3	6.5	11.2	7.2	1.8	5.5	18.6	4.5	9.5	10.9	7.0
Support Personnel	5.3	9.3	2.2	28.4	1.8	7.4	6.1	5.9	4.6	13.1	2.2	3.2	4.6	7.3	8.8	6.6	3.7	5.9
Management and Supervision	1.3	3.0	0.1	0.0	0.8	1.2	2.2	1.6	1.0	2.4	0.9	1.0	1.1	2.5	1.5	1.2	1.5	1.3
Total unit cost	100	100	100	100	100	100	100	100	100	100	100	100	100	100	100	100	100	100

All figures are in percentages except those in the first row, which are in 2012 US$ and PPP-adjusted to the sub-Saharan Africa region.

The second largest component of unit costs was consumables, accounting for 28% of the total, on average, across all 99 facilities. Again there was significant variation within countries, with the highest percentage devoted to consumables in Uganda (50%), followed by a relatively high percentage in Namibia (48%). While Uganda's relatively higher percentage spent on consumables was offset by relatively lower personnel costs, Namibia experiences relatively higher costs for both personnel and consumables, where in-service training costs are included in salary costs. The percentages for consumables for three countries grouped around the average—Tanzania (30%), South Africa (29%), and Kenya (27%)—while Zambia (at 18%) has the lowest percentage devoted to consumables.

Capital costs were the fourth largest contributor to overall unit cost, with an average of 10% spent across all facilities. The most significant outliers were a relatively higher amount in Zambia (23%), while the lowest amount was in Namibia (0%) where no data were collected for that category. The relatively higher costs in Zambia can be attributed to certain pieces of medical equipment that were associated exclusively with scaling up VMMC (e.g., autoclaves and diathermy machines).

The fifth largest component of unit costs, on average, was maintenance and utility costs at 7% of the total unit cost. Two countries were significant high outliers in this category—South Africa (24%) and Zambia (19%), while both Namibia and Uganda had relatively lower percentages in this category, at 2% each. In Zambia, high maintenance and utility costs appeared to be due to relatively higher electricity costs, while in South Africa, the relatively lower direct personnel costs could imply higher cost shares for other components.

Support personnel accounted for approximately 5% of total unit costs; South Africa was a significant outlier with over 28% of its total unit cost devoted to support personnel. The higher percentage of cost for support personnel in South Africa (as with maintenance and utility costs) may be due to the relatively lower share direct personnel costs have in the total unit cost due to the use of task sharing. Three other countries were fairly close to the overall average for support personnel: Kenya (9%), Uganda (7%), and Zambia (6%). Both Namibia and Tanzania had lower percentages devoted to support personnel (2%), which is in contrast to the relatively higher amount attributed to direct personnel costs.

Management and supervision costs accounted for an even smaller percentage than support personnel costs, with an average of slightly more than 1% for all countries, ranging from a high of 3% in Kenya to a low of 0.1% in Namibia. Management and supervision costs were not collected in South Africa.

Aggregated labor-related component costs within countries—direct personnel, training, support personnel, and management and supervision—did not vary as much across countries as the individual components. Overall, the average total labor-related costs were 48% of the total unit costs, with the highest percentage in Tanzania (60%), followed by Kenya (57%), Namibia (50%), Uganda and South Africa (43%), and finally Zambia (41%). Thus the highest and lowest observations range within about 20% of the average in each direction; that is, the percentage in Tanzania—60%—is 20% higher than the average of 48%, while the percentage in Zambia is 17% lower than the average of 48%, implying a tighter distribution than when the components are examined individually.

Turning to examining the unit cost components according to other characteristics, unit costs did not vary substantially according to urban/rural status, and in fact appeared to be higher in rural areas: the average unit cost was US$47 in urban areas, while the average unit cost was US$57 in rural areas. Rural areas paid relatively more for direct personnel costs (44% versus 38% for urban), and slightly more for capital costs (7% versus 5%) and training costs (11% versus 8%). Urban areas, on the other hand, paid relatively more for consumables: 32% versus 26% for rural areas. The relatively higher personnel and training costs for rural areas are primarily driven by the results from Tanzania, as fewer procedures were performed; this is discussed in detail in a paper in this collection [6].

The absolute unit cost was significantly higher for public service providers (US$65), almost twice as much as for NGO service providers (US$36), and more than twice as much as for private service providers (US$26). Private service providers spent relatively more on consumables (49% versus 28% to 30% for the other two service provider categories), while public service providers spent more on direct personnel costs (43% versus approximately 33% to 34% for the other two service provider types). NGO service providers had relatively higher maintenance and utility costs (11% versus 7% and 2% for public and private service providers, respectively) and relatively higher support personnel costs (13% versus approximately 2% to 3% for the other service providers). However, with only 10% of service providers classified as private, some small-sample bias might be present.

As expected, the absolute unit cost for hospitals was slightly higher than that for health centers: US$53 versus US$47. Personnel costs accounted for a higher percentage of the total unit cost for hospitals, accounting for 42% versus 31% for health centers, while health centers had a higher percentage devoted to expenses for maintenance and utilities, 19% versus 6% for hospitals. Note that, although the unit cost for dispensaries was higher even than that for hospitals, there were only six dispensaries in the dataset, and the results seemed to be skewed by two facilities with high personnel costs.

Finally, we examined the components of unit cost according to service delivery mode. Recall that the majority of the sites in this sample (78%) were fixed/static sites, and that service delivery through outreach was sampled only in Kenya (Nyanza) and Tanzania, while service delivery through campaigns was sampled only in Tanzania. With these caveats in mind, the unit cost for campaigns relative to all other service delivery modes across all other countries was the highest, with a unit cost of US$70, followed by a unit cost for outreach of US$55, and finally the lowest unit cost for fixed/static sites, US$45. Note that the outreach unit cost included the outlier from the island outreach site in Tanzania, which in adjusted terms is US$198. The fixed/static sites spent a larger proportion on consumables, 33% versus about 23% and 24% for the other two service delivery modes, while the outreach and campaign modes had larger expenses for direct personnel, 47% and 44%, respectively, versus 37% for fixed/static sites.

### Econometric results

Table 3 highlights the results of the econometric analysis, where the reference variables were rural location, private provider, hospital facility, and Kenya. Of the key cost-scale variables of interest, six variables—volume of VMMCs (including volume, volume squared, and volume cubed), total labor cost, consumable cost, maintenance and utility cost—showed significant p-values less than 0.05. However, the coefficients for these variables represented a small proportion of total cost—volume (0.04%), total labor cost (0.2%), consumable costs (0.4%), and maintenance and utility cost (0.3%) only. Although NGO service providers, public service providers, health centers, dispensaries, and outreach accounted for more than 1% of the total cost, these values were not statistically significant.

**Table 3:** Cost function estimate.

	Coefficient	Robust Standard Error	P-value
Constant	1.927568	0.04396	0
Volume	0.000461	0.000042	0
Volume^2^	−0.000000143	1.83E-08	0
Volume^3^	1.22E-11	1.84E-12	0
Prevalence of Male Circumcision	−0.00051	0.000505	0.317
Total Labor Cost	0.002789	0.000507	0
Consumable Cost	0.004339	0.001389	0.002
Maintenance and Utility Cost	0.003324	0.000667	0
Urban	0.003889	0.011389	0.733
NGO Service Provider	0.019043	0.022677	0.401
Public Service Provider	0.027506	0.022549	0.223
Health Center	−0.01225	0.013138	0.351
Dispensary	0.020336	0.012379	0.1
Outreach	0.011413	0.013611	0.402
Campaign	−0.00789	0.025773	0.759
Namibia	−0.22291	0.048898	0
South Africa	−0.06832	0.035021	0.051
Tanzania	−0.10329	0.034536	0.003
Uganda	−0.00994	0.021406	0.643
Zambia	−0.00223	0.026449	0.933

Source: Authors' calculations.

In terms of non-scale factors, the regression results showed that, even after costs are adjusted to a regional average, costs vary by country; unit costs in Namibia and Tanzania were lower than those in Kenya by 22.3% and 10.3%, respectively.

**Figure 1:**
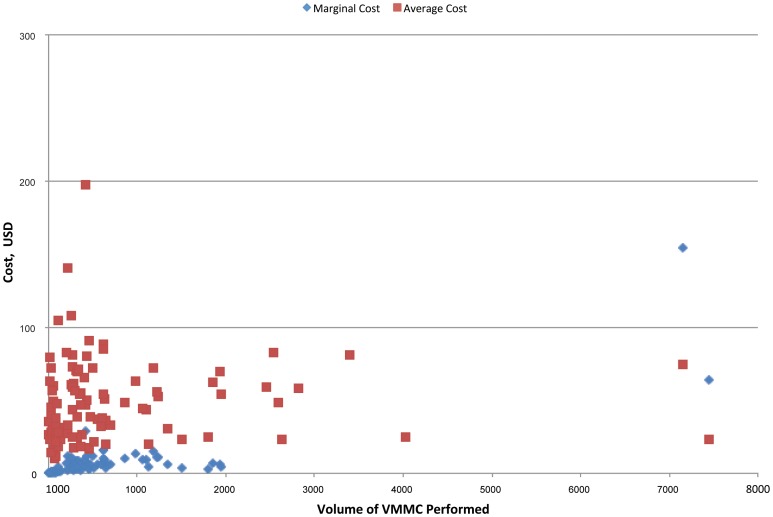
Predicted average and marginal cost of VMMC programs. Red square: Average cost. Blue diamond: Marginal cost.

The marginal cost of the volume of VMMCs performed, evaluated at the median, was $6.46 or 0.04% of the median total cost (see Figure 1). In Figure 1, with the volume of VMMCs performed plotted against marginal and average costs, predicted marginal cost is seen to decrease as the volume of VMMCs performed increases, with marginal cost at the 75th percentile of volume of VMMCs performed approximately two-thirds the marginal cost at the 25th percentile ($5.10 and $7.55, respectively). This pattern confirms that, on average, these facilities are on the downward-sloping section of the marginal cost curve, implying that minimum efficient scale has not yet been reached, and further efficiencies are possible.

**Figure 2:**
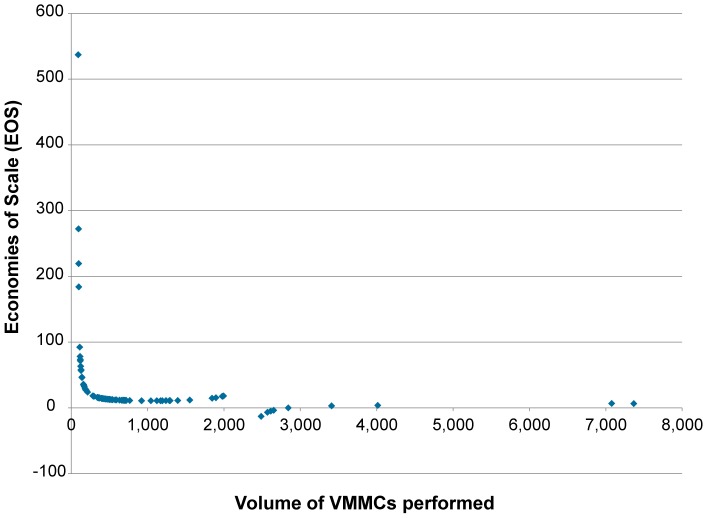
Predicted economies of scale of VMMC programs. Blue diamond: predicted economies of scale.

The EOS for the median volume of VMMCs performed was calculated at 8.14, also indicating that improvements in efficiency could occur. As Figure 2 shows, EOS falls from 23 at the 25th percentile volume of VMMCs performed to 5.3 at the 75th percentile. The scale of the fall in EOS as VMMC volume increases, as well as the shape of the marginal cost curve, suggests that we are still at the ascension phase of the scale-up of VMMC, where continuing to add new sites results in additional start-up costs as well.

### Limitations of the analysis

The inherent nature of the data available for this study predisposes them to the following limitations:

- Although every attempt was made to ensure appropriate sampling of facilities in each of the countries, these results may not be representative of VMMC costs across the selected countries, or of VMMC costs for HIV prevention as a whole.
- This analysis was based on a cross-sectional data set rather than a panel data set, so could not include time-related effects such as lagged cost or coverage.
- Demand creation costs were not included in this analysis because of the inherent challenges in isolating demand creation costs for VMMC, as well as the difficulties in identifying what would be an appropriate level of spending on such activities. It should be noted, however, that demand creation spending can have a significant impact on overall unit costs, given that greater demand is likely to create greater economies of scale.
- Countries did not necessarily categorize personnel in a similar way across countries. For example, if a nurse offered VMMC services but also offered support services, he/she was generally categorized as either a full-time direct staff or as full-time support personnel. As a result, the distinction between direct personnel and support personnel could lead to the disparate results observed in countries like Tanzania and South Africa.

The cost function estimated does not differentiate between variable and fixed costs, that is, all costs are treated as variable costs, and as such the results may be more relevant for longer-term planning, when all costs become variable. Note, however, that the results above derived from the smaller sample size, which show minimal differences between the calculated EOS when capital costs are included and excluded, imply that these results can be used for policymakers in a relatively short-run time horizon.

## Discussion

In order to make effective decisions about how to allocate limited resources to HIV prevention measures—of which VMMC shows substantial positive impact—it is crucial that efficiency improvements be identified and leveraged to reduce the overall cost of the procedure. The analysis in this paper suggests that there is significant room for efficiency improvement by identifying increasing marginal cost, stagnant average cost, and falling EOS as VMMC volume increases. In particular, the scale of the fall of EOS indicates that services are still very much at the scale-up phase where program investment costs and low patient volume continue to contribute to increased cost per patient receiving VMMC. Additionally, the most recent data on VMMC coverage—84% in Kenya, 57% in Namibia, 35% in South Africa, 72.3% in Tanzania, 26% in Uganda and 15% in Zambia— indicate that half of the sample countries included in this analysis have yet to meet national targets for VMMC coverage [28]. More specifically, this suggests that the volume of men seeking VMMC is too low to reach the point at which cost efficiencies set in. This finding suggests that more needs to be done to create demand for VMMC. Preliminary data from Tanzania suggest the advantages of offering VMMC as part of a campaign. At campaign sites in Tanzania, an average of 2,890 circumcisions were conducted as opposed to an average of 738 at fixed/static sites. The average weighted cost for a circumcision conducted at a campaign site was $420 less than the average weighted cost of $738 at fixed/static sites. This finding suggests that campaigns increase volume and decrease unit cost of VMMC, and thus offer a venue to increase the volume of VMMC services and achieve EOS efficiencies.

In looking at the key cost drivers, personnel and consumables stand at 36% and 28%, respectively, indicating areas where efficiency gains can be made. A key aspect of improving efficiency in personnel costs is task sharing VMMC procedures. Efficiency improvements in consumables are likely to occur over time when commodities are combined into a disposable surgical kit and as prices and distribution costs decrease. The adoption of many of the elements of the MOVE model, particularly in South Africa, shows promising practices for implementing changes to improve efficiency.

Before implementing these changes, however, it is important that additional analyses be conducted where national level and long-term cost data are included in the overall cost calculations, as well as cost data for demand creation. For example, as noted by Bertrand et al. [29], the correlation between the quantity of resources required for demand creation at a national level and the subsequent actual uptake of services is extremely difficult to predict. Nonetheless this limitation should not be underestimated because countries do expend significant resources on creating demand for VMMC services. In addition, further data from other facilities within these countries, as well as from facilities in other countries, would provide further robustness to the estimates. These additional data will likely identify additional areas for improving cost efficiency and provide governments and funders with guidance on how best to invest resources in expanding access to VMMC.

Finally, since the time of data collection (2008–2011) was relatively early in VMMC scale-up in each of the countries included in this sample, the costs associated with this study may not be representative of current costs associated with VMMC provision.
